# Suppression of the µ Rhythm during Speech and Non-Speech Discrimination Revealed by Independent Component Analysis: Implications for Sensorimotor Integration in Speech Processing

**DOI:** 10.1371/journal.pone.0072024

**Published:** 2013-08-22

**Authors:** Andrew Bowers, Tim Saltuklaroglu, Ashley Harkrider, Megan Cuellar

**Affiliations:** 1 Department of Communication Disorders**,** University of Arkansas, Fayetteville, Arkansas, United States of America; 2 Department of Audiology and Speech Pathology**,** University of Tennessee Health Science Center, Knoxville, Tennessee, United States of America; University of Milan, Italy

## Abstract

**Background:**

Constructivist theories propose that articulatory hypotheses about incoming phonetic targets may function to enhance perception by limiting the possibilities for sensory analysis. To provide evidence for this proposal, it is necessary to map ongoing, high-temporal resolution changes in sensorimotor activity (i.e., the sensorimotor *μ* rhythm) to accurate speech and non-speech discrimination performance (i.e., correct trials.)

**Methods:**

Sixteen participants (15 female and 1 male) were asked to passively listen to or actively identify speech and tone-sweeps in a two-force choice discrimination task while the electroencephalograph (EEG) was recorded from 32 channels. The stimuli were presented at signal-to-noise ratios (SNRs) in which discrimination accuracy was high (i.e., 80–100%) and low SNRs producing discrimination performance at chance. EEG data were decomposed using independent component analysis and clustered across participants using principle component methods in EEGLAB.

**Results:**

ICA revealed left and right sensorimotor µ components for 14/16 and 13/16 participants respectively that were identified on the basis of scalp topography, spectral peaks, and localization to the precentral and postcentral gyri. Time-frequency analysis of left and right lateralized µ component clusters revealed significant (*p*FDR<.05) suppression in the traditional beta frequency range (13–30 Hz) prior to, during, and following syllable discrimination trials. No significant differences from baseline were found for passive tasks. Tone conditions produced right µ beta suppression following stimulus onset only. For the left *µ*, significant differences in the magnitude of beta suppression were found for correct speech discrimination trials relative to chance trials following stimulus offset.

**Conclusions:**

Findings are consistent with constructivist, internal model theories proposing that early forward motor models generate predictions about likely phonemic units that are then synthesized with incoming sensory cues during active as opposed to passive processing. Future directions and possible translational value for clinical populations in which sensorimotor integration may play a functional role are discussed.

## Introduction

It is well known that the acoustic speech signal does not directly map onto perceived speech-sound categories. This phenomenon is known as a ‘many-to-many’ mapping between acoustic correlates and phonemic units. In other words, different acoustic cues may be associated with the same phoneme and a single acoustic cue may be perceived differently depending on the surrounding speech sounds [1], [2]. For example, although listeners identify the consonant/d/as the same phonemic category for the syllables/di/and/du/, the formant transitions characterizing the consonants are different for the two syllables [2]. Despite the complex relationship between acoustic features and perception, humans successfully process speech even when acoustic cues are mixed with background noise. As such, the process by which categorical percepts are recovered from variable acoustic cues has been long been a matter of debate and is known as the ‘lack of invariance problem’ [3]–[11].

To many theorists, the invariance problem suggests that acoustic cues alone cannot specify what humans perceive as distinct phonemic units [4], [12]–[16]. Early gestural theories addressed the invariance problem by suggesting that the goal of speech perception was not to perceive acoustic cues but instead to recover invariant articulatory gestures from the acoustic signal. Liberman’s motor theory proposed that acoustic cues were perceived as the invariant, intended articulatory targets that give rise to the acoustic signal, linking perception and production via a specialized phonetic module [4]. Alternatively, Fowler’s direct realist theory proposed that sensory properties (i.e., acoustic cues) could directly specify invariant articulatory gestures without reference to intended articulatory targets [12]. Although the details of the two theories differ, both imply that the motor system is critical for perception and that direct mapping between sensory signals and invariant gestures is a solution the problem of invariance [6]–[8]. However, a number of findings are inconsistent with the prediction that the motor system is necessary for perception, including categorical perception in non-human species [18], lack of motor system activation in some speech processing tasks [8], [9], [19]–[22], and lesion evidence suggesting only subtle deficits with damage to the speech motor system [22]. As such, although gestural theories propose a solution to the problem of invariance, they have been widely debated from their inception.

In contrast with the prediction that the motor system is necessary for speech perception, recent theories of speech processing have proposed that sensorimotor simulations may play a role in aiding acoustic analysis of speech depending on the perceptual environment and goals of the perceiver [6], [7], [11], [23], [24]. These theories appear to have been influenced by Helmholtz’s concept of constructivism and suggest that previous experience derived from speech production (i.e., the motor system), multisensory information (e.g., visual mouth movements), and language may function to disambiguate cues derived from the acoustic speech signal [11]. As such, ‘constructivist’ accounts propose that accurate perception is achieved via an active process in which hypothesized interpretations of the incoming speech signal weight sensory perception toward expected acoustic features in a manner similar to ‘analysis-by-synthesis.’ [6]. Under conditions in which acoustic cues are ambiguous, Stevens and Halle’s theory long ago proposed that the articulatory system might generate hypotheses about the intended articulatory target that can then be synthesized with incoming acoustic features [23].

Despite decades of controversy, there is little neurophysiological evidence clearly favoring constructivist accounts of speech processing over gestural theories of perception [11]. This is no small matter as the primary goal of speech perception theory and decades of psychoacoustic research has been to account for the invariance problem. Due to predictions about the process by which invariance is achieved, constructivist and direct-realist perspectives make clear, divergent predictions about when sensorimotor integration should occur relative to the onset of acoustic stimuli. Constructivists predict that motor regions should be active prior to and following the arrival of acoustic speech signals (i.e., hypothesis and test). Direct realists predict motor activation during acoustic stimulation only (i.e., direct mapping). Further, gestural theories in general (i.e., direct mapping theories) propose that the motor system should be active in all listening conditions, whereas constructivist theories propose that the motor system will be active depending on the perceptual environment and goals of the perceiver [6]. A resolution to these fundamental questions may favor one computational mechanism over another and thus may also be critical to developing models that can accurately account for speech perception in natural environments (i.e., face-to-face interaction) [23]–[32].

Neurophysiological theories proposing a role for sensorimotor integration have suggested neural mechanisms following gestural and constructivist theories. On the gestural side, the discovery of sensorimotor ‘mirror neurons’ in the F5 area of the macaque, a proposed homologue for the premotor cortex (PMC) near Broca’s area in humans, has renewed interest in direct-realism and motor theory [11], [28]. As in direct realism, it has been suggested that the PMC might support a direct action to observation matching system based on the property of neural identity [16] or alternatively might support the recovery of intended articulatory goals from the sensory signal in a manner similar to motor theory [11], [28]. In this way, the PMC may function as a multimodal way station for directly linking incoming perceptual signals with the neural commands that generated those signals (i.e., direct mapping theories) [25]. As such, the human mirror neuron system (MNS) pivoting around the PMC has been taken as physiological support for long held notions intrinsic to gestural theories [16], [26].

In contrast with purely gestural notions, a number of neurophysiological frameworks have proposed that online sensorimotor simulations for speech processing may play a functional role in some contexts [6]–[11]. Although theories diverge on some details, including whether or not the MNS is involved, most accounts suggest that motor regions cooperate with sensory regions in the temporal and inferior parietal lobes known as the dorsal auditory stream network [9]–[11], [26]–[30]. During language development, the dorsal stream may allow infants to translate speech sounds stored in the temporal lobe into motor commands that generate those same sounds in frontal regions, allowing for intimate connections between learned auditory targets and the motor commands required to produce them [33], [34]. During receptive speech processing in some contexts (e.g., speech in noise), it is thought that the same neural architecture involved in speech production may be recruited for perception [24]. Some authors have speculated that motor regions may function in a predictive capacity similarly to the manner in which attention is thought to operate in visual perception [7], [10], [11]. According to these notions, forward internal models (i.e., predictions) generated via dorsal stream motor regions might initiate sensory constraints on incoming acoustic information as in ‘analysis-by-synthesis’ [6]–[8], [10], [11], [35]–[37]. These forward models may function by applying gain to sensory neurons tuned to expected features, by suppressing neurons tuned to irrelevant features, or perhaps using both processes at once [10]. As such, during active processing forward articulatory models of the motor system predict activity in motor regions prior to acoustic onset (i.e., articulatory hypotheses) and immediately following sensory analysis (i.e., test). This prediction is orthogonal to the predictions of direct-realism in which sensory properties directly specify invariant speech gestures (i.e., during sensory analysis only) and occur irrespective of attention to the signal.

In support of a role for the motor system in perception, a number of neuroimaging studies have demonstrated that brain regions classically involved in speech production are also active during perception. In passive perception tasks, in which participants are not required make decision about auditory stimuli, neuroimaging studies have shown variable activation in motor regions across tasks [6]–[8], [9]–[11], [38]–[40]. However, in active tasks activity in motor regions has been shown to be related to task performance. In a functional magnetic imaging study (fMRI), Binder et al. [40] demonstrated that blood-oxygen level dependent (BOLD) activity in the PMC/Broca’s area increased as performance decreased in a two-forced choice (e.g.,/ba/vs./da/) speech perception-in-noise task, suggesting that the motor system may play a compensatory role with increases in environmental noise. Callan et al. [41] found greater activity in the PMC for perception of second language contrasts relative to native language contrasts that also increased as participants learned new contrasts. Transcranial magnetic stimulation (TMS) studies in which motor regions are systematically disrupted have provided further support for a functional role. Meister et al. [42] found that disrupting the ventral premotor cortex (vPMC) resulted in a decrease in phonetic discrimination in a speech in noise task. D’Ausillio [43] found that stimulation to the precentral gyrus (primary motor cortex; M1) resulted in the facilitation of phoneme identification for effector involved. Sato et al. [44] found that disruption of a region within the precentral gyrus resulted in decreased reaction time only when segmentation (e.g.,/bat/vs./rim/) as opposed to discrimination (e.g.,/bad/vs/dad/) was required, suggesting that the motor system might have some function related to more difficult tasks requiring greater demands on working memory. Those findings are also consistent with previous imaging studies showing increased BOLD activation in motor regions for a similar task [45].

Although neuroimaging and TMS studies implicate the motor system in perception, they have contributed little clear physiological evidence addressing how it functions. In order to test contrasting theories, it is critical to demonstrate that ongoing motor activity prior to and immediately following acoustic onset is related to perceptual performance. Recently, Callan et al. [11] addressed this gap in a two-forced choice, speech-in-noise design in which brain activity was recorded using both fMRI and high-temporal resolution magnetoencephlography (MEG). Activation in the PMC was associated with changes in spectral power that were greater for correct relative to incorrect trials in the time-periods prior to and following acoustic input, strongly supporting constructivist theories of speech processing. However, as that study did not employ non-speech control stimuli, it remains possible that early and late activity in the PMC may function in a broader capacity. The PMC near Broca’s area is known to be active in a number of tasks, including rapid pitch discrimination [46] and tone segmentation tasks [47], suggesting that areas of motor system might also function for rapid perceptual judgments or rapid auditory processing. Additionally, if early motor activity is specifically related to speech perception accuracy, it has the potential to be used as a brain computer interface (BCI) approach to improve subsequent performance in populations with processing deficits. As fMRI and MEG are prohibitively expensive for use in many clinical settings, there is a need to develop a less expensive alternative for BCI approaches. Deficits or differences in networks for sensorimotor integration for both speech perception and production have been implicated in a number of communication disorders, including autism, language impairment, hearing impairment [48], stuttering [49], and aphasias [10], all of which are commonly treated in speech and hearing clinics.

Perhaps another method by which to quantify ongoing, high-temporal resolution activity in the motor system is to measure event-related spectral perturbations (ERSPs) of the sensorimotor *μ* rhythm using relatively low-cost scalp-recorded electroencephalography (EEG). A large corpus of evidence has demonstrated that event-related descynchronization (ERD) (i.e., power decrease or suppression) of the sensorimotor *μ* rhythm occurs during the observation, imagination, and execution of biological movements, strongly suggesting the *µ* is a functional correlate of movement processing [50]–[66]. The *µ* rhythm of the EEG is composed of at least two dominant frequency components, one occurring at ∼10 Hz and another occurring at ∼20 Hz. The ∼10 Hz component tends to be localized to the somatosensory cortex and the ∼20 Hz component is known to emerge in a somatotopic manner from the precentral gyrus corresponding with the motor cortex for the effector involved (e.g., lip vs. hand movements) [63]–[65]. As the rhythm is localized to the sensorimotor cortex, it is thought to be a ‘down-stream’ measure of motor activity emerging from the PMC [65]. Two relatively recent studies have demonstrated sensorimotor suppression (i.e., electrode locations C3, Cz, C4) to visual and audiovisual speech signals [61], the continuous presentation acoustic syllables in noise, and during segmentation tasks in ideal listening conditions, suggesting that sensorimotor rhythms may suppress as a function of auditory stimulus characteristics [66]. However, to test theoretical predictions, it is critical to demonstrate that suppression time-locked to stimulus events prior to and following stimulus onset is related to perceptual performance (i.e., correct trials).

Recent studies have demonstrated that the sensorimotor and other traditional EEG components can be isolated from other volume conducted components of the EEG via a blind source signal separation (BSS) approach known as independent component analysis (ICA). A BSS approach to signal analysis allows for an examination of temporal dynamics of EEG components with minimal influence from other components (i.e., the influence of volume conduction). As the aim of the current study is to measure millisecond resolution ERSPs prior to and following a speech and rapid pitch discrimination task to shed light on the functional role of the motor system without the influence of other sources, EEG data were decomposed in the current study using ICA and subsequent within subject clustering [67], [68]. As in previous neuroimaging studies, the current study employs classical two forced-choice speech and non-speech (i.e., rapid pitch change) in noise design in which EEG time-frequency changes are measured before, during, and following stimulus onset in both passive listening and active listening tasks. The specific aims of the present study are to: 1) identify independent components (IC’s) consistent with known features of the sensorimotor *µ* rhythm; and 2) investigate ERSPs for *µ* rhythm components relative to stimulus type (i.e., speech relative to non-speech), onset relative to acoustic input, and discriminability (i.e., correct versus chance trials). The second aim was achieved by examining the spectra, source estimations of independent component clusters, and the time-course of ERSP activation patterns. If the motor system is critical for perception as predicted by gestural theories, it should be active in passive as well as active syllable discrimination tasks. However, if the motor system functions as internal models predict, activity prior to and immediately following acoustic input would be expected for active discrimination trials that is greater for correct trials over those identified at chance. By contrast, if the motor system functions as predicted by direct realism, only activity during stimulus processing would be expected.

## Methods

### Experimental Design

The current proposal employs a classical two forced-choice speech and non-speech (i.e., rapid pitch change) perception design similar to that used in previous studies [11], [40]. In addition to addressing theoretical predictions, steps were taken in the current design to address concerns that have traditionally limited interpretation of neuroimaging evidence of motor activity in sublexical, two-forced choice tasks. It has been suggested that activity in the motor system may be more related to covert rehearsal (i.e., internal speech production) to enhance working memory or alternatively due to sensory-decision mechanisms as opposed to perception per se. In the current study, this possibility was addressed in two ways. First, as motor processing in passive tasks is thought to reflect covert rehearsal (i.e., after stimulus onset) and motor processing in active tasks may play a functional role (prior to and after stimulus onset), both passive and active tasks were employed. Covert rehearsal was also addressed via the temporal resolution of the EEG. If covert rehearsal or working memory for the response was responsible for activity in motor regions, such activity would not be expected prior to an acoustic stimulus. Further, a minimum of 200 ms would be required for participants to process an auditory stimulus and to initiate covert rehearsal [11]. As such, if differences between correct and chance trials are found immediately following stimulus offset, it is unlikely that such differences would be due to covert rehearsal.

Second, in two forced-choice paradigms in which a same/different decision is required, it has been suggested that activity recorded within classical speech production areas might arise from sensory-decision processes [10], [11], [40]. To address this possibility, two active discrimination tasks (i.e., requiring a sensory-decision via button press) for speech and non-speech auditory stimuli were presented at high and low signal-to-noise ratios (SNRs) such that a large number of correct trials and chance trials were submitted. As participants can still detect auditory stimuli at lower SNRs, if suppression of the sensorimotor rhythm were related to sensory-decision only as opposed to stimulus processing, no differences between correct trials and those discriminated at chance levels would be expected. In other words, as sensory-decision mechanisms are not different between the high and low SNR conditions, no differences would be expected. In addition, no differences between correct speech and rapid-pitch discrimination tasks would be expected as sensory decision requirements were similar. Further, no differences in lateralization (i.e., left relative to right hemisphere motor activity) would be expected for speech and control non-speech auditory stimuli since for both an active decision was required.

### Participants

Sixteen right-handed English-speaking adults (15 female and 1 male) with a mean age of 25 (range 20–42) participated in this study. Participants were recruited from the general population at the University of Tennessee. Participants reported no diagnosed history of communicative, cognitive or attentional disorders. Degree of handedness was assessed using the Edinburg Handedness inventory [69]. This study was approved by the Institutional Review Board of the University of Tennessee Health Science Center. Prior to the experiment, all participants were provided with an informed consent document approved by the Institutional Review Board and all participants gave written informed consent prior to inclusion.

### Stimuli

**Figure 1:**
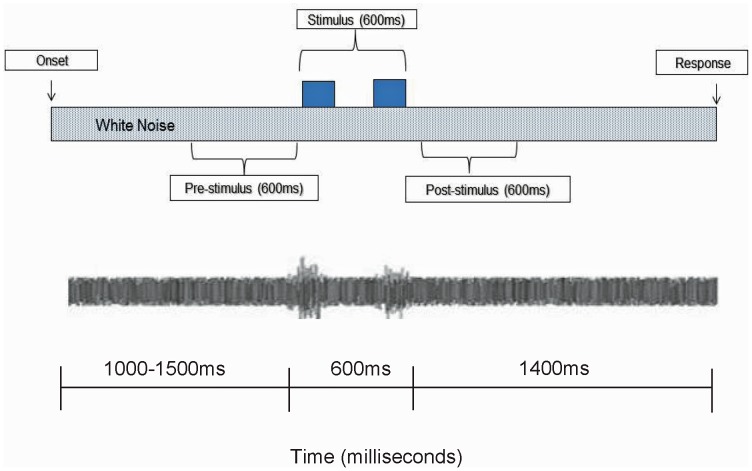
Sample time-line of one trial with time periods of interest prior to, during, and following stimulus onset.

Speech stimuli consisted of/ba/and/da/syllable generated using AT&T naturally speaking text-to-speech software. The software generates syllables from text using speech synthesized from a human male speaker. Half of the stimuli were composed of different initial sounds (e.g.,/ba/and/da/) and the other half were the same (e.g.,/ba/and/ba/). The stimuli were normalized to have the same root-mean-square (RMS) amplitude and low-pass filtered with a cutoff at 5 kHz. Each stimulus syllable was 200 ms in duration with an interstimulus interval of equal length (i.e., 200 ms). Thus, the total time required to present a stimulus pair was 600 ms. For the tone discrimination task, sine-wave tone sweeps were generated using a procedure adapted from a previous neuroimaging study [46]. Tone-sweep stimuli were composed with an 80 ms modulated tone onset and a 120 ms steady state 1000 Hz sine-wave. As for the speech stimuli, tone-sweeps were generated, low-pass filtered with a cut-off at 5 kHz, and normalized to have the same RMS amplitude as the speech stimuli. Tone pairs differed only in whether the pitch onset was lower at 750 Hz than the steady state tone or higher at 1250 Hz. For both speech and tones the time between trials (i.e., intertrial interval) was 3000 ms. White noise for the tone and speech stimuli was generated and processed using the same procedure as for the speech sounds, with a low-pass filter cut-off at 5 kHz. All auditory stimuli were processed using Soundtrack Pro academic software on an iMac (2 GHz intel core duo) computer and were sampled at 44 kHz. Conditions were placed in random order prior to presentation. All stimuli were presented at an absolute intensity of ∼70 dB. An example time line of one stimulus trial is displayed in Figure 1.

Previous investigations have shown better than chance performance on a forced choice syllable discrimination task using a +4 dB SNR and chance performance using a −6 dB SNR [11], [40]. However, pure tones may be detected with noise intensities as high as 18 dB above pure tone intensity (i.e., −18 dB SNR) [70]. To account for differences in perceived loudness between tone and speech stimuli, preliminary behavioral data was collected on 10 female participants using Stim2 presentation software presented through Etyomotic ER1–14A tube phone inserts in a sound treated booth. Syllable and tone stimuli were embedded in white noise and presented in 20 trials using the time scheme shown in Figure. 1 at the following SNRs −18 dB, −12 dB, −6 dB, +4 dB. Syllable stimuli were identified above chance in the +4 db condition only. Accuracy for tone- sweep conditions were not above chance in −18 dB SNR, with 60% in −12 dB SNR, 78% in the −6 dB condition, and 76% in +4 dB condition. Paired-t tests revealed no significant difference (*p*>.05) between the +4 dB and −6 dB tone-sweep conditions. As such, the SNRs for the syllables were set at +4 dB and −6 dB and for tone-sweeps at +4 dB and −18 dB.

### Procedure

Stimuli were presented using Stim 2 4.3.3 stimulus presentation software on a PC computer. The experiment was conducted in an electronically and magnetically shielded, double-walled, sound-treated booth. Participants were seated in a comfortable reclining armchair with their heads and necks well supported. Participants were told that they would be listening to white noise, syllables, and tones. They were instructed that the onset of one trial would commence when white noise was audible, followed by either syllable or tone stimuli. Participants were asked to indicate whether the syllables or tone-sweeps sounded the same or different by pressing a button using the left thumb only. Premotor planning for repeated finger movements has been shown to occur ∼1 second prior to muscle contraction [63], [71] and sensorimotor suppression to peak briefly (∼200 ms) following a manual response to a perceptual target [72]. To further control for the possibility that preparation for the response might confound motor activity related to stimulus processing, participants were signaled to respond via a 100 ms, 1000 Hz sine wave tone 1400 ms after stimulus onset. To control for stimulus-response bias in the button press task, the order of the button press was counterbalanced [11].

All conditions were randomized prior to presentation and presented in two randomized blocks consisting of 40 trials each. Performance was evaluated as a percentage of correct trials (%CT) and response time (RT). Participants were asked to listen under the following conditions: 1) Passively listening to noise (PasN); 2) Passively listening to speech syllables in +4 dB noise (PasSp+4 dB); 3) Passively listening to tone-sweeps in +4 dB noise (PasTn+4 dB); 4) Active syllable discrimination-in +4 dB noise (ActSp+4 dB) 5); Active tone-sweep discrimination-in +4 dB noise (ActTn+4 dB); 6) Active syllable discrimination in −6 dB noise (ActSp−6 dB); 7) Active tone-sweep discrimination in −18 dB noise (ActTn−18 dB).

### Data Acquisition

Thirty-two electrode channels were used to acquire EEG data based on the extended international 10–20 method of electrode placement [73] using an unlinked, sintered NeuroScan Quik Cap. Recording electrodes included Cz, C3, C4, CP4, CP3, Pz, P3, P4, P8, Fz, F3, F4, F7, F8, FC4, O1, O2, FP1, FP2, FT7, FT8, T3, T4, T5, T6, TP8, TP7 with two electrodes on the left (M1) and right mastoids (M2). The reference electrode was placed on the nasion and the ground electrode was at Fpz. The electro-oculogram (EOG) was recorded by electrodes placed on the left superior orbit and the left inferior orbit (VEOG) and on the lateral and medial canthi of the left eye (HEOG) to monitor vertical and horizontal eye movements, respectively. The impedances of all electrodes were measured at 30 Hz before, during, and after testing and were never greater than 5 KΩ.

EEG data were collected using Compumedics NeuroScan Scan 4.3.3 software and the Synamps 2 system. The raw EEG data was filtered (0.15–100 Hz), and digitized via a 24-bit analog-to-digital converter at a sampling rate of 500 Hz. Data was time-locked to the onset of individual speech perception trials. After data collection, the recorded EEG signal and electro-oculogram (EOG) data was segmented into single trials lasting approximately 5000 ms each, spanning from −3000 ms to +2000 ms with reference to stimulus onset (i.e., zero time). That is, time prior to syllable and tone-sweep stimuli was considered negative and time following syllable and tone-sweep stimuli was considered positive. To examine pre and post-stimulus activity, the EEG data was epoched into 5000 ms segments. EEG data were visually inspected and trials contaminated by gross artifacts greater than 200 µV were removed. A minimum contribution of 40 epochs for each participant in each condition was required for inclusion in the experiment. Due to a contribution of only 20 trials in several conditions, one participant was omitted from analysis.

### ICA Preprocessing

As in previous studies using both ICA decomposition and sLORETA source localization analysis, to decrease computational requirements for ICA processing, data were digitally resampled using a cubic spline interpolation method and a division of 1024 Hz (here 256 Hz) adequate for investigating all frequencies up to the gamma frequency range [74], [75]. Prior to ICA training, EEG data were concatenated for each participant across conditions. Subsequent ICA training was implemented using the extended *runica* algorithm in EEGLAB [76]. The initial learning rate was set to.001 with a stopping weight of 10–7. Linear decomposition using the extended Infomax algorithm was conducted for each participant across experimental conditions. The algorithm spheres or decorrelates the data matrix prior to ICA rotation and computes the variance of IC projection weights on to the original EEG channel data [67]. The resulting square weight matrix (30×30) is thus applied to each participant, yielding a single set of weights for each experimental condition expressing independence in the data. This process allows for a comparison of condition differences for the same set of component weights. The inverse weight matrix (W^−1^) can then be projected onto the original EEG channel configuration, providing a spatial scalp topography for the components.

Independent components (IC’s) were evaluated for each participant across experimental conditions using three criteria. First, an automated algorithm (ADJUST) shown in a previous study to have good inter-rater reliability with researchers experienced in IC noise removal, was used to tag non-brain artifact components in the EEGLAB module [77]. Scalp-maps and log spectra were also visually inspected for indicators of non-brain artifact including high power in low frequencies, abnormal spectral slope, and scalp-topographic distributions known to be associated with eye-movement and temporal muscle contraction [11], [67]. ICs with characteristic signs of non-brain artifact were then pre-tagged for each subject. Second, IC’s with 20 trials having outlier values (µV SD set to 10) over the electrode with maximum power were eliminated [11]. Finally, equivalent current dipole (ECD) models for each component were computed using a four-shell spherical head model (BESA) in the DIPFIT toolbox [78], freely available at sccn.ucsd.edu/eeglab/dipfit.html). Standard 10–20 electrode coordinates were warped to the BESA head model followed by automated coarse and fine-fitting to the spherical wire matrix, yielding dipole models for each of 480 ICs. The procedure involves hypothesizing a dipole source that could have generated the scalp potential distribution for a given IC and then computing the forward model that explains the highest percentage of the variance in the scalp map. ECD modeling is well-suited to explaining IC scalp topographies as they can be modeled efficiently with a single dipole source representing synchronous activity within a small patch of cortex.

### sLORETA Source Estimations

sLORETA is a functional imaging technique that provides standardized linear solutions for modeling 3-D distributions of the likely cortical generators of EEG activity [79]. The software uses a 3-D spherical head model separated into compartments including, the scalp, skull, and brain. sLORETA analysis operates under the assumption that scalp-recorded signals originate primarily in the cortical gray matter/hippocampi and that neighboring neurons are synchronously activated, giving rise to a signal that is distinct from surrounding noise. The head model is standardized with respect to the Talairach cortical probability brain atlas, digitized at the Montreal Neurological Institute (MNI) and uses EEG electrode coordinates derived from cross-registrations between spherical and realistic head geometry [80]. The brain compartment includes 6,239 voxels (5 mm resolution). Electrode coordinates were converted to ASCII text format and exported to sLORETA from the EEGLAB module. For each IC, inverse ICA weight projections onto the original EEG channels were exported to the sLORETA data processing module for each participant. Cross-spectra were computed and mapped to the standard Taliarach brain atlas cross-registered with the Montreal Neurological Institute (MNI) coordinates, yielding sLORETA estimates of current source density for each of 480 ICs.

### Independent Component Clustering

To identify similar independent components across participants, 480 (30×16) components were then clustered using measure product methods in the K-means statistical toolbox implemented in EEGLAB [81]. The toolbox uses principle component clustering methods to reduce data dimensions and yields similar component clusters across participants. Here, 28 possible component clusters were considered. The data dimensions were reduced to 10 with the standard deviation set to 3. As such, ICs more than 3 standard deviations from any cluster mean were excluded as an outlying cluster. As the sensorimotor *μ* rhythm is known to have characteristic peaks at ∼10 and ∼20 Hz and source locations within the sensorimotor cortex, scalp-maps, log spectra, and equivalent current dipole models were precomputed and used in the clustering analysis. Component power spectra for each subject were calculated by averaging fast Fourier transform (FFT) spectra for each epoch using a window length of 256 points. Scalp topographies were computed as 30 channel (x,y) map gradients and ECD models were precomputed in the manner described in a previous section. After clustering, only components with a single dipole model within the head volume accounting for 80% or greater of the variance in the independent component scalp distribution were included in *μ* component clusters. Pre-identified noise components tagged prior to the analysis were used to identify clusters accounting for non-brain sources. Only dipole locations and sLORETA source maximum voxels within the precentral or postcentral gyrus with spectral peaks ∼10 and ∼20 Hz were included in *μ* component clusters.

### Event-related Spectral Perturbations

To examine stimulus induced changes in the EEG spectrum, ERSP transforms were precomputed in the EEGLAB module using the STUDY command structure. ERSPs are changes scaled in normalized decibel units over a broad spectral range (here .5–40 Hz) [81]. For independent components, ERSPs are scaled in RMS decibel units on the same scale as the component. Thus, IC scalp map topographies and ERSPs share the same RMS scale in decibel units. In this study, ERSPs were computed using a Morlet sinusoidal wavelet set at 3 cycles at 3 Hz rising linearly to 20 cycles at 40 Hz. A 1000 ms pre-stimulus baseline was selected from the silent intertrial interval. This baseline served as a time period during which a surrogate distribution was generated. The surrogate data distribution is constructed by selecting spectral estimates or each trial from randomly selected latency windows in the specified epoch baseline. In this study, the baseline data was sampled 200 times, producing a baseline distribution whose percentiles were taken as significance thresholds [81]. Significant changes in spectral power (i.e., increases or decreases from baseline) were then tested using a bootstrap resampling method. Significant differences from baseline (*p*<.05 uncorrected) were considered in the subsequent within subjects analysis of ERSPs for identified *μ* component clusters.

Analysis of condition effects for the left and right *μ* ERSPs were carried out using the STUDY command structure in EEGLAB. The single trial current for all seven experimental conditions for frequencies between 3–40 Hz and times from −600 ms to 1500 ms post-stimulus onset were entered into a time-frequency analysis. For the two conditions in which performance was better than chance (ActSp+4 dB and ActTn+4 dB) only trials discriminated correctly were considered in the ERSP analysis. A mean of 64 trials across conditions were entered into the ERSP analysis. Wavelet estimates across trials for each time and frequency were then converted to a time-frequency matrix (69×105) from 3.4 Hz to 39.9 Hz to −589 to 1441 ms. To test the significance of condition effects, non-parametric random permutation statistics in a 1×7 repeated measures ANOVA design were computed. The advantage of using non-parametric statistics for hypothesis testing of ERSPs is that this approach does not assume that the data are normally distributed. As discussed in previous papers, the event-related spectral increases (ERS) and decreases (ERD) that characterize ERSPs are frequently non-normal [71]. Random permutation methods generate a surrogate distribution constructed from time-frequency values randomly shuffled from each condition across all possible permutations. This random distribution represents the null hypothesis that no condition differences exist. In the current study, 2000 random permutations were computed and compared to *F*-values for the mean condition differences. To control for the inflation of Type I error rates associated with multiple comparisons, a correction for false-discovery rate (*p*FDR) was applied, allowing for a conservative test of condition effects [82].

## Results

### Percentage Correct Trials

**Figure 2:**
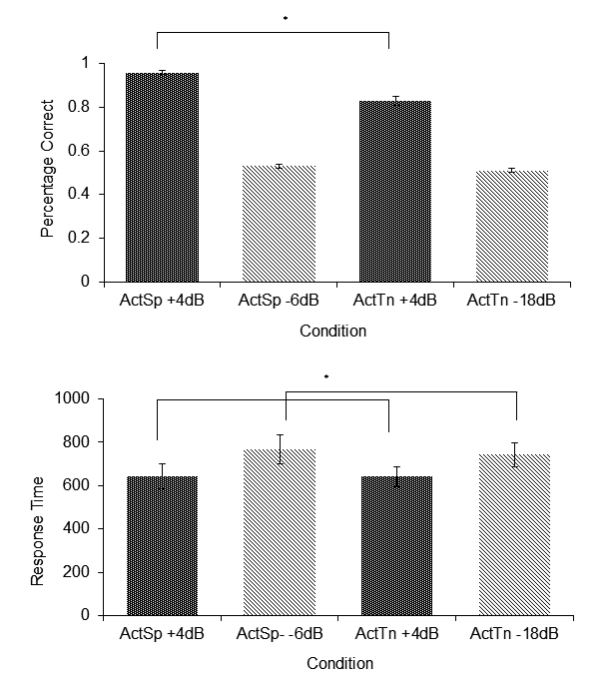
Means and standard errors for percentage correct trials and response time. A) active speech and tone perception conditions for percentage correct and B) active speech and tone perception conditions for response time. Significant condition differences at *p*<.05 are indicated by *.

The means and standard errors for percent correct trials (%CT) in the four active speech and tone perception conditions are displayed in Figure 2A. Prior to the analysis, trials with response times (RT) greater than three standard deviations from the mean response time (i.e., trials greater than 1996 milliseconds) were removed and were not considered in any subsequent analysis. Performance on the active perceptual identification tasks (i.e., tasks in which a response was required) was assessed as a percentage of correct trials. Performance in the ActSp+4 dB condition was at near ceiling levels with a mean of 96% (SE = .01) correct. The ActSp+4 dB condition was associated with better performance than the ActTn+4 dB condition, with a mean of 83% (SE = .02) correct in the latter. The mean for the ActSp−6 dB and ActTn−18 dB were 52% (SE = .01) and 51% (SE = .01) correct respectively. For the active conditions, a repeated measures analysis of variance (ANOVA) for the factor condition (1×4) revealed a significant main effect [(*F* = (3)131.65, *p*<.01]. A series of planned, a priori orthogonal single degrees of freedom comparisons were employed to determine condition differences. A significant difference was found for a comparison between %CT in the ActSp+4 dB condition and the ActTn+4 dB condition [(*F* = 39, *p*<.01, *η* ^2^ = .72, *Φ* = 1]. No significant difference was found for a comparison of the Actsp−6 dB and Actn−18 dB conditions [(*F* = 1.79, *p* = .20]. The ActSp−6 dB and ActTn−18 dB were not significantly different from chance [*t* = .98, *p* = .20]. Thus, as expected, only the speech and tone-sweep conditions with a relatively high SNR (i.e. +4 dB) were associated with better than chance performance.

### Response Time

The means and standard errors for button press response time are depicted in Figure 2B. Response times (RT) were evaluated as the time in milliseconds from the cue to respond (i.e., 1000 Hz tone) to the button press response. RTs for each subject in the four active conditions were entered into a repeated measures ANOVA with the factor condition (1×4). The analysis revealed a significant main effect for condition [(*F* = 3.71, *p*<.01, *η* ^2^ = .19, *Φ* = .77]. A series of planned, single degrees of freedom a priori contrasts revealed significant differences between correct trials in the ActSp+4 dB and ActTn+4 dB compared to chance trials in the ActSp−6 dB and ActTn−18 dB conditions respectively [(*F* = 7.23, *p*<.01, *η* ^2^ = .32, *Φ* = .71]. No significant difference was found between the Actsp+4 dB and ActTn+4 dB conditions [(*F* = .00 *p* = .96] or between the ActSp−6 dB and ActTn−18 dB [*F* = .24 *p* = .62]. The mean RT for the two conditions in which performance (ActSp+4 dB and ActTn+4 dB) was above chance were 642 ms (SE = 58) and 641 ms (SE = 47) respectively. The mean RT for the two conditions in which performance was at chance levels was 767 (SE = 68) for speech and 743 ms (SE = 55) respectively. Taken together, the analysis of behavioral responses revealed an inverse relationship between perceptual performance in the active conditions and button press response time.

### Independent Component Clustering

Independent component clustering revealed eight distinct component clusters with neural as opposed to non-brain (i.e., artifact) sources. Six component clusters accounted for eye-blinks and vertical eye-movements, horizontal eye-movements, temporal muscle noise, and nonspecific noise (electromagnetic noise). Component clusters with similar scalp-topographies, spectra, ECD, and sLORETA CSD locations were found for a left hemisphere frontal, bilateral frontal midline cluster, central midline cluster, and left and right posterior temporal clusters. However, because the focus of the current investigation is on the sensorimotor *μ* rhythm, only left and right sensorimotor components are discussed further.

**Figure 3:**
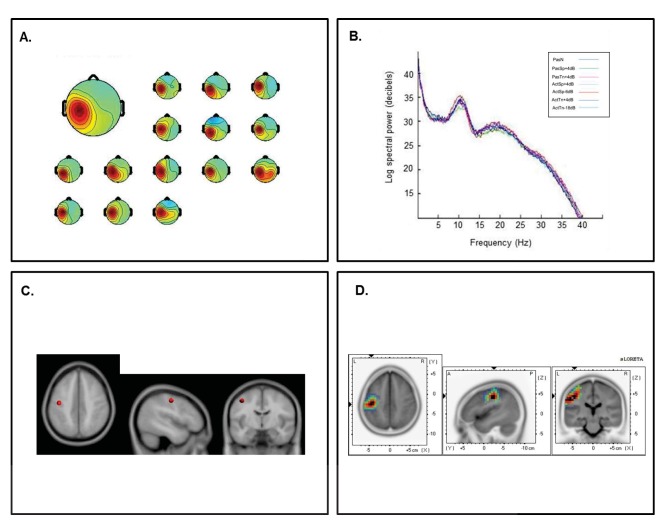
Cluster results for the left-hemisphere *µ* component. A) mean scalp potential distribution (W^−1^) scaled to RMS microvolts and individual scalp distributions for each participant, B) mean spectra of the component as a function of condition, C) average equivalent current dipole location, and D) maximum current source density voxels (*t*-values) with greater values in darker colors and smaller values in lighter colors (NIH Micro template) (at *p*<.001 corrected for multiple comparisons).

**Figure 4:**
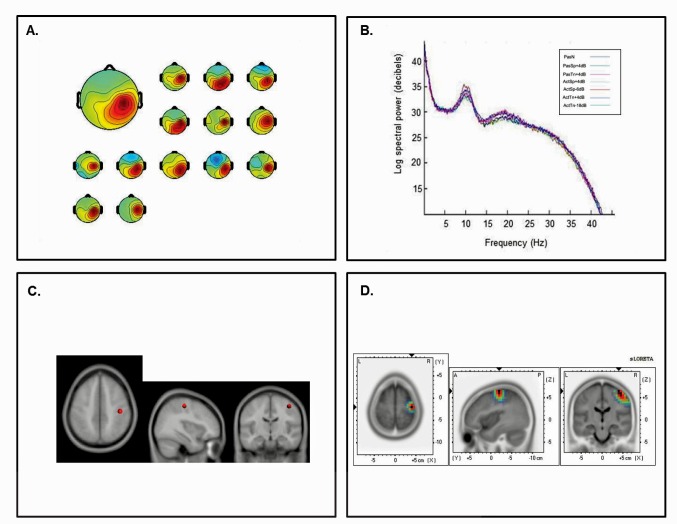
Cluster results for the right-hemisphere *µ* component. A) mean scalp potential distribution (W^−1^) scaled to RMS microvolts and individual scalp distributions for each participant; B) mean spectra of the component as a function of condition, C) average equivalent current dipole location, and D) maximum current source density voxels (*t-*values) with greater values in darker colors and smaller values in lighter colors (NIH Micro template) (at *p*<.001 corrected for multiple comparisons).

Fourteen participants submitted ICs with the hallmark characteristics of the left sensorimotor *μ* rhythm and 13 participants submitted ICs with hallmarks of the right *μ* rhythm. The left cluster revealed mean scalp-topographies centered over the left sensorimotor cortex (Figure 3A) whereas the right cluster showed a similar topography over the right hemisphere (Figure 4A). For both clusters, log spectra revealed distinct spectral peaks at ∼10 Hz and ∼20 Hz (Figures 3B and 4B) and ECD locations within the left and right pre or postcentral gyri with an average dipole location at Taliarach coordinates [(x,y,z) −42, −13, 47] in the left hemisphere and [(x,y,z) 41, −12,42] in the right hemisphere. The residual variance not explained by the single dipole model was 4.33% for the left hemisphere and 5.73% in the right hemisphere.

To evaluate the statistical significance of dipole locations across participants, statistical comparisons relative to zero (i.e., no activation) were computed for all *μ* scalp topographies in the sLORETA statistical module [83]. A paired t-test was carried out for frequencies between 0–40 Hz (159 frames) with the smoothing parameter set to 1 (single common variance for all variables), using 5000 random permutations yielding corrected *t*-value thresholds for all 6,235 voxels in the sLORETA solution space. The paired test revealed significant voxels at *p*<.001 in the precentral and postcentral gyri with maximum current source density estimates at Taliarach [*t* = 2.85 (x,y,z) −45, −18, 42] in the left hemisphere and Taliarach [*t* = 2.52 (x,y,z) 40, −16,61] in the right. As left hemisphere activity was of interest, to determine the extent to which sLORETA and ECD estimates were correlated, a bivariate correlation analysis was conducted on the maximum CSD coordinates and the ECD coordinates for each sensorimotor IC in the left hemisphere. The analysis revealed correlation coefficient of *r* = .93 that was significant at *p*<.01, suggesting a close relationship between CSD and ECD estimates. As such, topographic results and source localization estimates support the hypothesis that ICA captures synchronous EEG signals consistent with a small patch of cortex.

### Event-related Spectral Perturbations

**Figure 5:**
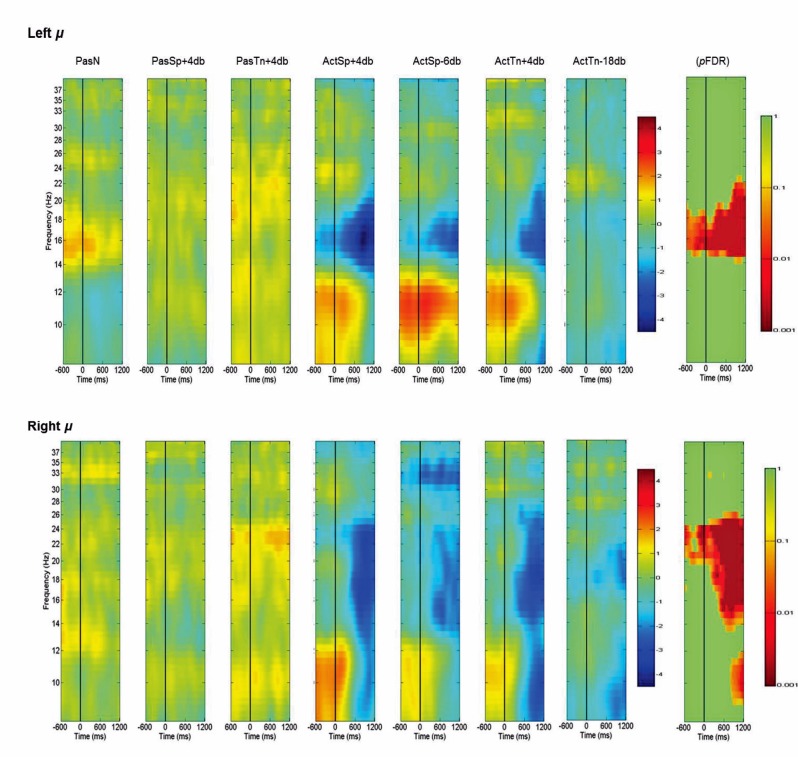
Mean left and right hemisphere *µ* time-frequency ERSPs (event-related spectral perturbations). ERSPs are scaled in the same root-mean-square decibel units as a function of condition (1×7) and random effects analysis indicating significant values in the traditional beta (13–30 Hz) and alpha ranges (8–13 Hz). Non-significant values are colored green, with significant values shown in orange and red. Event-related decreases in spectral power are indicated in blue (−4.5) and increases are indicated in red (4.5).

Left and Right *μ* mean ERSP values across subjects and conditions are shown in a time-frequency map with corrected significance values for condition in a separate map (Figure 5). Non-significant values are depicted in green and significant values are depicted in color from orange for weaker values to red for stronger values (*p*FDR<.10 to *p*FDR<. 001). A repeated measures ANOVA design with the factor condition (1×7) revealed no significant differences for the number of trials submitted between conditions (*F* = .92, *p* = .48). The initial permutation analysis (1×7) revealed significant ERSPs in the 15–20 Hz range (beta) the left *µ* component and the 15–25 Hz range for the right hemisphere component corrected across the entire time-frequency matrix (*p*FDR<.05; 69×105) (see Figure 5). Significant time-frequency values were found in the time-periods prior to, during, and after stimulus onset with a peak event-related decreases in spectral power (i.e., ERD) in the time period after stimulus offset. To determine the sources of condition effects, two separate ANOVA designs were computed using the STUDY command structure. Because the time periods before, during, and after stimulus onset were of interest, all subsequent analysis were based upon the time period from −600 ms to 1200 ms. In other words, as the total time to present a stimulus was 600 ms, all times and frequencies between 3 and 40 Hz for time periods 600 ms prior to until 1200 ms following the stimulus were investigated to test proposed hypotheses.

### Left *μ*

**Figure 6:**
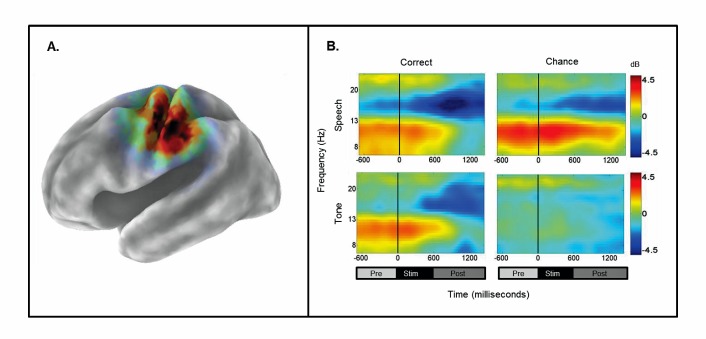
Mean ERSPs for correct and chance trials as a function of stimulus type and performance level for the left-hemisphere *µ* cluster. A) sLORETA solutions depicted on a 3D Van Essen average template; B) mean time-frequency ERSPs (event-related spectral perturbations) as a function of stimulus type (speech and tone) and performance level (correct and chance) for the time-periods prior to stimulus onset, during stimulus presentation, and after stimulus-offset.

First, to determine whether any significant differences existed between the PasN baseline and the other passive conditions, a 1×3 ANOVA was conducted for the PasN, PasSp+4 dB, and PasTn+4 dB conditions. For the left *μ*, no significant differences corrected across the entire time-frequency matrix (69×92) were found (*p*FDR>.05) in 15–20 Hz range, indicating no differences between the PasN baseline condition and the other two passive conditions. Analysis of the active conditions in which discrimination was required (1×4), revealed a significant main effect (*p*FDR<.05; 69×92) in the 15–19 Hz range for the time period between 600–1200 ms following stimulus offset. To assess which conditions were significantly different from the PasN baseline, a series of paired t-tests were performed. Significant differences (*p*FDR<.05; 69×92) for the time periods before, during, and after stimulus onset were found for the ActSp+4 dB and ActSp−6 dB only. A paired comparison between the ActSp+4 dB and ActSp−6 dB conditions across the 15–19 Hz range between 600–1200 ms period (*p*FDR<.05;8×31) revealed a significantly larger peak ERD in the ActSp+4 dB condition just following stimulus offset and lasting until 1100 ms. As such, the left component cluster showed significant effects for only the syllable discrimination task and further showed significant differences in the time period following stimulus offset for correct discrimination trials in the ActSp+4 dB condition relative to the chance trials in the ActSp−6 dB condition (Figure 6).

### Right *μ*

**Figure 7:**
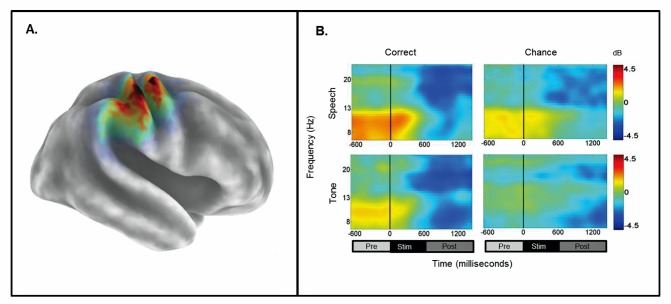
Mean ERSPs for correct and chance trials as a function of stimulus type and performance level for the right-hemisphere *µ* cluster. A) sLORETA solutions depicted on a 3D Van Essen average template; B) mean time-frequency ERSPs (event-related spectral perturbations) as a function of stimulus type (speech and tone) and performance level (correct and chance) for the time-periods prior to stimulus onset, during stimulus presentation, and after stimulus-offset.

The initial permutation analysis revealed significant ERSPs at *p*FDR<.05 in the 15–25 Hz range (beta) for the right *µ* component (Figure 5). Significant time-frequency values corrected across the entire time-frequency matrix (*p*FDR<.05; 69×92) were found in the time-periods prior to, during, and after stimulus onset with a peak event-related decrease in spectral power in the time period after stimulus onset. To determine the sources of condition effects, a 1× design for the passive conditions (PasN, PasSp+4 dB, and PasTn+4 dB conditions) was conducted. The ANOVA revealed no significant differences (69×92; *p*FDR>.05). Analysis of the active conditions in which a sensory-decision was required (1×4; ActSp+4 dB, ActSp−6 dB, ActTn+4 dB, and ActTn−18 dB), revealed no significant differences (*p*FDR<.05; 69×92). To assess which conditions were significantly different from the PasN condition (i.e., the baseline), a series of paired contrasts were performed. Significant differences (*p*FDR<.05; 69×92) for the time periods before, during, and after stimulus onset were found for correct trials in the ActS+4 dB and chance trials in the ActSp−6 dB conditions. For the tone-sweep conditions, significant suppression occurred only after stimulus onset (*p*FDR<.05; 69×92). Although ERDs were found for individual participants in the time-period prior to tone-sweep discrimination trials, overall results did not fall below the significance threshold. Thus, although active tone discrimination conditions differed from the passive noise baseline in the time period following stimulus onset, no significant differences were noted between the active conditions (Figure 7).

Previous studies have suggested that reaction time may provide a measure of sensory decision processes related to a response [40]. To investigate the possibility that ERSPs following stimulus onset might be related to the button press response, significant ERSPs values (i.e., 15–20 Hz for the left *μ* and 15–25 Hz for the right *μ*) were extracted from correct trials in the +4 dB condition and averaged over three time-periods of interest prior to, during, and following stimulus onset (−600–0, 0–600, and 600–1200). A bivariate correlation analysis was performed on each participant’s mean ERSP values in the time period following stimulus offset (600–1200) and RT for each subject in the left hemisphere component cluster. The results indicated no significant correlation between RT and ERSPs (*r* = .02 *p* = .94), suggesting little relationship between RT and left hemisphere sensorimotor desynchronization (i.e., suppression) where the main effects of ERD were related to task performance.

In summary, left and right *μ* rhythm clusters were associated with suppression relative to PasN during the active syllable discrimination task prior to, during, and following the onset of syllable stimuli. Peak suppression values prior to acoustic onset occurred between ∼200 and 100 ms in the syllable discrimination conditions, with the largest suppression values occurring just following acoustic offset (∼600–800 ms) in those conditions. Relative to chance trials, correct trials were associated with significantly greater suppression in the time period immediately following acoustic syllable stimuli only. Tone-sweep discrimination trials were not associated with suppression prior to stimulus onset relative to PasN. However, tone-sweep trials were significantly different from PasN in the time period after stimulus presentation only. Finally, no significant differences were found between correct tone-sweep discrimination trials and trials discriminated at chance.

## Discussion

To investigate the time-course of sensorimotor processing in speech and non-speech processing, the current study employed independent component analysis of event-related EEG to measure activity of sensorimotor *µ* rhythm. The aims of the current study were to first identify ICs with hallmark features of the sensorimotor rhythm and second to investigate suppression of the rhythm relative to task demands, stimulus type, and discriminability. To our knowledge, this is the first study to employ a BSS method to investigate suppression of the *µ* rhythm in a speech and non-speech discrimination task commonly employed in neuroimaging studies. As such, the present findings have implications for the measurement of EEG during auditory tasks. Taken together, findings provide evidence that the *μ* rhythm is involved in active syllable discrimination performance. Further, findings may have implications for potential use as a brain computer interface (BCI) approach in speech and hearing clinics. In the discussion following, each of the specific aims are addressed separately and subsequently framed within an overall discussion of the theoretical and possible clinical significance.

### Specific Aim 1

The first aim of the investigation was to identify ICs with hallmark features of the sensorimotor *μ* rhythm following ICA decomposition. As ICA is a blind source separation algorithm, components must be inspected for identifying features consistent with traditional elements of the EEG signal. Several findings for left and right hemisphere components are consistent with known characteristics of the *μ* rhythm. First, cluster analysis revealed left and right hemisphere IC clusters with topographic distributions over the sensorimotor cortex with spectral peaks at ∼10 Hz and ∼20 Hz and average dipole locations in the lateral portion of the central sulcus [84]–[89]. Second, a distributed localization approach indicated significant activations in the precentral and post central gyri, consistent with distributed source locations over the sensorimotor cortex within a small cortical patch [80]. Third, relative to the passive noise baseline, the left and right sensorimotor *μ* rhythms in the active discrimination conditions were associated with differential suppression in the traditional beta range (here 15–25 Hz), consistent with the activity of neuronal populations in the precentral gyrus [63]. These findings strongly suggest that component clusters with the hallmark characteristics of the sensorimotor *μ* rhythm were differentially involved in the processing of speech and non-speech auditory signals.

In previous studies, it has been demonstrated that ICA is capable of separating the most synchronous EEG signals associated with small patches of cortex (∼3 cm) [81]. As electrical potentials are limited by the speed at which they travel through a conducting volume, it is thought that ICA captures local field potentials within small cortical areas. In the present study, it appears that ICA captured activity over the lateral portion of sensorimotor cortex with dominant frequency components at ∼10 and ∼20 Hz. Although the ∼10 Hz and ∼20 Hz components of the *µ* are thought to be phase locked and thus interdependent, there is reason to suspect functional differences between the two. High-density MEG studies have shown power changes at ∼10 Hz associated with dipole locations near the somatosensory cortex bilaterally when band pass filtered to exclude the ∼20 Hz rhythm. Suppression of the ∼10 Hz rhythm is also associated with tactile stimulation and binocular rivalry [63], [85], suggesting that it is more generally associated with somatosensory activity. Further, with the administration of drugs effecting motor unit firing (e.g., diazepam), power centered near 20 Hz is doubled and 10 Hz activity is not affected [84]. As such, the most likely role for the ∼10 Hz rhythm during movement processing is in coding the somatosensory consequences of the perceived or performed movement when required [63]. In the present study, the ∼10 Hz rhythm was enhanced during active and passive processing relative to the silent recording interval, most likely reflecting cortical inhibition of the somatosensory cortex.

Several lines of evidence suggest that beta suppression (i.e., ∼20 Hz) over the sensorimotor region is generated in motor regions. First, MEG studies have shown that beta suppression during the overt production, imagination, and observation of movement is associated with dipole locations near the primary motor cortex bilaterally (BA4) following the moving body part (e.g., more lateral locations for mouth movements) [63]. Second, in agreement with a source in the primary motor cortex, ∼20 Hz suppression has also been obtained from intracranial recordings within the central sulcus [86]. Third, ∼20 Hz suppression is coherent with motor unit firing [86], [63] and is enhanced by the administration of benzodiazapines, a drug known to result in clumsy, poorly controlled movements [87], [63]. Finally, beta suppression between 15 and 30 Hz has been shown to be inversely correlate with BOLD increases the premotor and primary motor cortex [90]–[94], suggesting convergence between hemodynamic approaches and electrophysiological approaches measuring local field potentials.

The functional distinction between the ∼10 Hz somatosensory and ∼20 Hz motor component in the current study is also broadly consistent with previous neuroimaging studies finding motor activity during speech processing in the auditory modality without differential changes in somatosensory activity [95]–[97]. Neuroimaging approaches have implicated overlapping regions within the precentral gyrus for both speech production and speech perception. Wilson et al. [97] found overlapping peaks of BOLD activity in regions extending from the precentral gyrus (MNI x,y,z = −51, −11, 46) to the posterior bank of the central sulcus (MNI x,y,z = −45, −13, 34) of the left hemisphere for speech perception and production. Callan et al., [11] found peak voxels for correct discrimination trials over incorrect trials at (MNI x,y,z = −48, −6,14) within the superior portion of precentral gyrus. These regions are near the mean dipole location and maximum CSD locations in the current study. Differential beta suppression in this study is also consistent with a recent MEG study finding increased N100 amplitudes for syllable identification in noise localized to a region of interest within the precentral gyrus that was greater for active perception relative to passive perception conditions [98]. As such, the current finding of differential beta suppression localized to the sensorimotor cortex is consistent with both known features of the *µ* rhythm and activation of the motor cortex during speech processing.

### Specific Aim 2

A second aim of the present study was to investigate whether suppression of the *μ* rhythm differed as a function of stimulus onset, stimulus type, and discriminability. First, in accordance with expectations behavioral measures indicated that active task performance differed as a function of SNR. A higher percentage CT and shorter RT in high SNR speech and tone conditions (ActSp+4 dB and ActTn+4 dB) was found relative to low SNR speech and tone conditions (ActSp−6 dB and ActTn−18 dB). As expected, due to the drastically decreased quality of auditory information in the low SNR conditions, perceptual performance was not above chance levels. Further, consistent with previous investigations of syllable discrimination in noise [11], [40], increases in RT were associated with decreases in accuracy. This finding is consistent with the notion that continuous sensory decision processes require greater time when a given decision is ambiguous relative to when it is more easily identified [40].

Second, suppression of the *μ* rhythm differed as a function of performance level for the syllable discrimination task only. Early activity (i.e., prior to stimulus onset) was present bilaterally for speech stimuli regardless of subsequent perceptual performance, suggesting sensorimotor processing prior to stimulus onset consistent with a kind of phonological selective attention. Third, brief peak activity (i.e., suppression) was significantly larger for the syllable discrimination task only when compared to extremely degraded stimuli (i.e., chance trials), indicating that although the sensorimotor cortex is active for both types of stimuli it is greater when auditory features are sufficient to specify phonemic units following sensory analysis. Fourth, significant suppression of the sensorimotor rhythm in the right hemisphere occurred for control tone stimuli in the time period following stimulus onset only. Thus, although speech stimuli elicit early activity peaking after stimulus discrimination, non-speech auditory stimuli are associated with activity following stimulus offset only and in the right hemisphere only. Further, as no differences were found between correct and chance tone-sweep discrimination trials, it is unlikely that right sensorimotor suppression was critical for the performance of that task. Thus, it would appear that the sensorimotor cortex in the left hemisphere was recruited for perceptual analysis in the syllable discrimination tasks only.

In addition to addressing theoretical predictions regarding the time-course of sensorimotor activity relative to acoustic stimuli, the experimental paradigm and findings were intended to address concerns that have traditionally limited findings of sensorimotor activity in neuroimaging studies including sensory-decision and covert rehearsal. First, significant differences for stimulus type and performance level for tasks in which an active decision was required were found. As the button press and sensory decision requirements were the same for correct speech and tone-sweep trials, no significant difference in suppression or in laterality would be expected for the two types of stimuli if sensory decision mechanisms alone accounted for differences. Another way in which sensory-decision processes and covert rehearsal were addressed was via the temporal resolution of the EEG. As beta suppression occurred prior to, during, and immediately following stimulus onset covert rehearsal does not easily explain the findings. Further, because significant differences between correct and chance trials began less than 200 ms (at ∼100 ms) following stimulus offset and lasted only 400 ms, it is unlikely that this early activity was due to covert production [11]. Finally peak suppression for both speech and tone stimuli following stimulus offset was brief, occurring within a 400 ms span of time and diminishing thereafter. If covert speech production for holding percepts in working memory accounted for suppression, peak suppression would be expected to be sustained until the sensory decision rather than diminishing prior to the response.

It is also worth noting that the experimental effects cannot be explained as simply preparatory activity for the upcoming button press response task. Although beta rhythms are associated with movement execution (and observation), it is unlikely that left hemisphere peak suppression here was related to preparation for the manual response for the following reasons: 1) There was no significant difference between baseline (PasN) and either active tone-sweep condition (AcTn+4 or AcTn−18) in the left hemisphere, indicating that preparation for a manual response cannot account for observed left hemisphere differences in the active syllable discrimination task; 2) Contrary to what would be expected for motoric execution, peak suppression was found over the hemisphere (left hemisphere) ipsilateral to the effector (left thumb); 3) Peak suppression occurred briefly following acoustic offset, more than 300 ms prior to the cue to respond and showed evidence of rebound thereafter; 4) Given that significant suppression prior to stimulus onset for the syllable discrimination conditions only, more than 2 s prior to the response, it is unlikely that preparation for the manual response can account for condition effects. For these reasons, we believe that it would be difficult to explain the left hemisphere differences between active correct and active chance trials as preparation for the manual response.

Whereas sensory-decision and covert-rehearsal do not easily explain the experimental outcomes, findings in the present study may be readily explained within the context of constructivist, internal model proposals of speech processing. More specifically, early beta suppression localized to the sensorimotor cortex prior to syllable discrimination is consistent with an early forward model that instantiates a general prediction about likely incoming sensory signals. As the subsequent percepts are unknown, such an anticipatory model may be explained as neural tuning to expected acoustic features of the upcoming stimulus [10], [11], [36]. Further, because early activity constitutes a prediction, continuous activity in the motor system to update the model would be expected until the initial set of articulatory hypotheses can be compared with online acoustic analysis. Peak activity just following acoustic analysis of the signal would be expected in the time period during which relevant features of the acoustic signal are matched with the initial forward constraints. Further, peak suppression would be expected only when the acoustic signal was sufficiently robust to be compared to the initial hypotheses. The finding of significant differences in *μ* suppression between correct and chance trials immediately after stimulus offset may be explained by such a process. In other words, it appears that early motor models may be instantiated when discrimination is required but fail to specify phonemes when auditory information is insufficient for comparison with the initial motor model.

However, given that internal articulatory models are thought to be developed via past experiences with producing speech, early activity would not be expected for non-speech auditory signals as those signals have not been repeatedly associated with vocal production. Indeed, early activity prior to stimulus onset was not found for correct or chance tone-sweep trials. As such, suppression of the sensorimotor rhythm following tone processing in the right hemisphere may be an attempt to internally simulate pitch changes using cortical representations of the vocal tract [47]. It is worth noting that this notion would predict forward models for not only speech sounds but for any sound repeatedly associated with actions for producing the same sounds [99]. Consistent with this notion, a number of studies have demonstrated motor activation for sounds associated with motor sequences required to produce them [100]–[102]. As such, to further investigate the role of the motor system and perhaps forward internal models in general auditory perception, future investigations may employ learning tasks in which pitch change stimuli are paired with actions required to produce them.

It is also worth noting that the present results favoring internal model concepts fit well with those designed to explain event-related decreases in spectral power prior to a range of cognitive tasks. These early spectral changes have been associated with subsequent performance in memory, attention, and visual tracking [103]–[109]. It has been suggested that multiple EEG sources oscillating at near harmonics of thalamic rhythms (∼10 Hz) may reflect inhibitory filter mechanisms mediating top-down attentional control. Power decreases are thought to signify a cortical release from inhibition that serves to ready the system for the coding of incoming information [106]. According to theories of EEG generation (e.g., local/global theories), transient global coherence between multiple cortical generators may instantiate top-down anticipatory processes that facilitate subsequent sensory processing [65]. In addition, recent theories of speech processing propose that ‘active sensing’ may be helpful to processing speech in natural, noisy environments and predict low-frequency (1–8 Hz) entrainment (i.e., phase coherence) across multiple brain regions during active processing. As such, the current results are likely to reflect just one component of a distributed neuronal network (e.g., the dorsal auditory stream) exerting top-down control on subsequent perception [27]. Future analyses may focus on how changes in ongoing beta rhythms are related to increases in phase coherence at low frequencies across component clusters.

### Conclusions and Future Directions

The current study is, to our knowledge, the first to employ a BSS method used previously in EEG studies of visual perception [81] and auditory event-related potential (ERP) paradigms [110] to address theoretical predictions about how the motor system functions in speech processing. The present study favors internal model frameworks of speech processing over mechanisms proposed by direct-realism. In addition, the study provides evidence supporting claims that these internal models operate similarly to a kind of phonological or articulatory selective attention [10], [11], [7]. For example, the finding that both correct and chance syllable discrimination trials were preceded by early *µ* suppression is what would be expected if forward articulatory models function similarly to selective attention. That is, if early articulatory hypotheses function in a manner similar to attention, early motor activity would be expected regardless of subsequent correct or chance level performance.

The study also provides further evidence that early forward models are related to perceptual performance at the point in time when acoustic features are sufficient for comparison with initial hypotheses (i.e., immediately following acoustic stimuli) in a manner similar to ‘analysis-by-synthesis.’ Furthermore, this study suggests that internal models are specific to a syllable discrimination task relative to a similar, rapid pitch discrimination task. As both tasks required attention to the task for successful discrimination, general attentional mechanisms cannot account for differences in early *μ* rhythm suppression for speech as opposed to tone-sweep stimuli. This finding is critical to determining underlying mechanisms, as to our knowledge no studies have measured the ongoing time-course of sensorimotor activity for speech and non-speech control tasks with similar attentional requirements. As such, results favor articulatory mechanisms that are either specific to speech [4] or alternatively to any auditory signal previously paired with vocal tract actions [47]; [111].

However, because specificity of motor regions in speech processing has traditionally been another area of heated debate, this issue deserves more explicit comment. The motor theory of speech perception postulated the use of articulatory goals to mediate and constrain perception long ago [4], [11]. According to Liberman’s ‘motor theory’, the lack of invariance problem is solved via a specialized phonetic module evolved to track intended invariant articulatory targets as opposed to acoustic features. The motor theory predicts that this articulatory phonetic module is critical for speech perception and thus that motor activity should be present in all contexts. However, as stated previously, a number of empirical findings are at odds with that prediction [26]. The lack of strong motor activity in passive speech perception in this study is consistent with some previous neuroimaging studies and is somewhat contrary to the predictions of the motor theory.

Although beta suppression during passive listening was not observed here, it is important to note that other studies have demonstrated activation of motor regions during passive listening tasks. Wilson et al [97] demonstrated motor activity during an auditory bombardment paradigm in which syllable stimuli were repeated in stimulus trains relative to non-speech listening tasks. However, a number of other neuroimaging studies have not shown motor activation during passive listening [10], [11]. One possible explanation for conflicting results is that the task and goals are important considerations in whether or not the motor system is recruited for task performance. Hickock et al [10] suggest that some passive tasks may induce covert rehearsal of repeated stimuli and propose that motor activity in active discrimination tasks may, by contrast, induce the use predictive internal model mechanisms. Callan et al [11] propose that passive conditions likely recruit predictive mechanisms to a lesser extent than tasks requiring active attention to speech stimuli, a conclusion similar to that reached by others [17]. In line with this point of view, a recent review has more specifically implicated beta suppression in efferent predictive mechanisms mediating active auditory perception [110]. As such, while the present study indicates sensorimotor activity specific to a syllable discrimination task, it also favors a more dynamic concept of speech processing in which sensorimotor integration via a transiently interactive neuronal system may function to aid acoustic analysis depending on context and perceiver goals. As such, speech processing may be characterized as one example of an ‘embodied’ process sharing a close connection between offline processing and the online sensorimotor mechanisms that give rise to those processes in real-world environments [113].

As early changes in spectral power are thought to influence subsequent perceptual performance in noisy conditions, it is likely that some populations with difficulty resolving percepts in noise might use sensorimotor integration in a compensatory capacity. Individual differences in motor activation have been shown to correlate with individual working memory capacity, suggesting a compensatory role when working memory is compromised [114]. Older adult populations without hearing loss have been shown to recruit areas of the motor system and frontal regions thought to be involved in attention and memory more heavily than younger listeners [115]. Dysarthria is a group of motor speech disorders that often significantly decreases the intelligibility of speech for both naïve and regular communication partners. Given that dysarthrias cause distortions of the consequent acoustic signal, it is possible that sensorimotor integration plays a compensatory role in perceptual learning for communication partners of those with dysarthria [116]. The motor system may function similarly to aid perception in children who have deficits in the perception of speech or spectotemporal analysis generally but have intact speech production. It is an open question whether the articulatory system plays an important role in perception for populations with hearing impairment. Further as deficits or differences in sensorimotor integration may play an important role in specific language impairment and autism spectrum disorders [48], the current methodology may provide information important for assessment and treatment in those populations. Establishing the ongoing time-frequency features of the EEG for both neurotypical and clinical populations addresses an important gap in the knowledge base as these signals have been shown to change as a function of experience and may thus be used as neuromodulatory feedback to enhance current therapeutic protocols [11].

Although these findings present evidence that the *μ* rhythm plays a functional a role in the speech processing, several limitations must be considered. First, as this study used only meaningless syllable and tone-sweep stimuli in the auditory modality only, the current findings are limited in the extent to which they can be generalized to other contexts. The motor system is likely to be more heavily involved in face-to-face interaction, during which gestural sources of information (e.g., facial and manual gestures) are known to influence perception [30]. Further, the current findings do not suggest that the motor system causes successful discrimination performance. In order to delineate the relative roles of multiple brain regions involved in speech processing, combined TMS and high-time resolution approaches are warranted [117]. Second, as only 32 channels were used in this study, it may be important to investigate whether improved spatial resolution may be obtained for high density electrode arrays (i.e., 64–128 channels) using ICA decomposition. However, informal comparisons between smaller and larger arrays have generally shown similar independent components associated with the most synchronous activity of the EEG and high correlations between dipole models for low density arrays and BOLD activity have been demonstrated [81], [118]. Third, although findings from this study implicate the sensorimotor rhythm in speech processing, performance was manipulated at the extremes so that performance was either accurate or not better than chance. It may be important to examine whether early and late sensorimotor activity tracks performance across different performance levels.

Limitations notwithstanding, findings in the present study help to flesh out the underlying role of early articulatory simulations in sublexical discrimination tasks. The presence of early beta suppression prior to stimulus onset and throughout stimulus processing would suggest that the motor system was involved at all stages of processing for speech stimuli, both for conditions in which percepts were discriminable and for conditions in which they were not. Thus, it appears that forward models may participate in perception even when the acoustic signal is so impoverished that discrimination between phonemes is not possible. Further, it appears that this process is specific to incoming acoustic signals that have been paired previously with articulatory movements. In light of the current findings, early forward models may function similarly to effort in attention. That is, when stimuli are attended regardless of the likelihood of success or failure, early motoric models are instantiated followed by an attempt at synthesis. However, synthesis or sensorimotor integration is achieved only when subsequent acoustic cues are sufficient to specify phonemic units.

## References

[1] Liberman AM, Delattre PC, Cooper FS, Gerstman LJ (1954) The role of consonant-vowel transitions in the perception of the stop and nasal consonants. Psychological Monographs: General and Applied 68: 1.

[2] Liberman AM, Delattre P, Cooper FS (1952) The role of selected stimulus-variables in the perception of the unvoiced stop consonants. The American journal of psychology 65: 497–516. 12996688

[3] Liberman AM, Mattingly IG (1985) The motor theory of speech perception revised. Cognition 21: 1–36. 4075760 10.1016/0010-0277(85)90021-6

[4] Liberman AM, Whalen DH (2000) On the relation of speech to language. Trends in Cognitive Sciences 4: 187–196. 10782105 10.1016/s1364-6613(00)01471-6

[5] Liberman AM, Cooper FS, Shankweiler DP, Studdert-Kennedy M (1967) Perception of the speech code. Psychological review 74: 431. 4170865 10.1037/h0020279

[6] Skipper JI, Nusbaum HC, Small SL (2005) Listening to talking faces: motor cortical activation during speech perception. Neuroimage 25: 76–89. 15734345 10.1016/j.neuroimage.2004.11.006

[7] Skipper JI, Nusbaum HC, Small SL (2006) Lending a helping hand to hearing: another motor theory of speech perception. Action to language via the mirror neuron system: 250–285.

[8] Skipper JI, Van Wassenhove V, Nusbaum HC, Small SL (2007) Hearing lips and seeing voices: How cortical areas supporting speech production mediate audiovisual speech perception. Cerebral Cortex 17: 23–87. 10.1093/cercor/bhl147 PMC2896890 17218482

[9] Hickok G, Poeppel D (2004) Dorsal and ventral streams: a framework for understanding aspects of the functional anatomy of language. Cognition 92: 67–99. 15037127 10.1016/j.cognition.2003.10.011

[10] Hickok G, Houde J, Rong F (2011) Sensorimotor Integration in Speech Processing: Computational Basis and Neural Organization. Neuron 69: 407–422. 21315253 10.1016/j.neuron.2011.01.019 PMC3057382

[11] Callan D, Callan A, Gamez M, Sato M, Kawato M (2010) Premotor cortex mediates perceptual performance. NeuroImage 51: 844–858. 20184959 10.1016/j.neuroimage.2010.02.027

[12] Fowler CA (1996) Listeners do hear sounds not tongues. J Acoust Soc Am 99: 3. 10.1121/1.415237 8819862

[13] Fowler CA An event approach to the study of speech perception from a direct-realist perspective. Journal of Phonetics 14: 3–28.

[14] Fowler CA, Brown JM, Sabadini, Weihing J (2003) Rapid access to speech gestures in perception: Evidence from choice and simple response time tasks. J Mem Lang 49: 396–413. 20622982 10.1016/S0749-596X(03)00072-X PMC2901126

[15] Skipper JI, Goldin-Meadow S, Nusbaum HC, Small SL (2009) Gestures orchestrate brain networks for language understanding. Current Biology 19: 661–667. 19327997 10.1016/j.cub.2009.02.051 PMC3767135

[16] Iacoboni M (2005) Understanding others: Imitation, language, empathy. Perspectives on imitation: From cognitive neuroscience to social science 1: 77–99.

[17] Skipper JI, Goldin-Meadow S, Nusbaum HC, Small SL (2007) Speech-associated gestures, Broca’s area, and the human mirror system. Brain Lang 101: 260–277. 17533001 10.1016/j.bandl.2007.02.008 PMC2703472

[18] Kuhl PK (1990) The special-mechanisms debate in speech research: Categorization tests on animals and infants. Categorical perception: The groundwork of cognition: 355.

[19] Hickok G (2001) Functional anatomy of speech perception and speech production: psycholinguistic implications. J Psycholinguist Res 30: 225–235. 11523272 10.1023/a:1010486816667

[20] Hickok G, Poeppel D (2007) The cortical organization of speech processing. Nature Reviews Neuroscience 8: 393–402. 17431404 10.1038/nrn2113

[21] Hickok G (2009) The functional neuroanatomy of language. Phys Life Rev 6: 121–143. 20161054 10.1016/j.plrev.2009.06.001 PMC2747108

[22] Lotto AJ, Hickok GS, Holt LL (2009) Reflections on mirror neurons and speech perception. Trends Cogn Sci 13: 110–114. 19223222 10.1016/j.tics.2008.11.008 PMC2921844

[23] Stevens KN, Halle M (1967) Remarks on analysis by synthesis and distinctive features. Models for the Perception of Speech and Visual Form. Cambridge, MA: MIT Press.

[24] Tremblay P, Small SL (2011) On the context-dependent nature of the contribution of the ventral premotor cortex to speech perception. Neuroimage 57: 1561–1571. 21664275 10.1016/j.neuroimage.2011.05.067 PMC3405555

[25] Gallese V, Gernsbacher MA, Heyes C, Hickok G, Iacoboni M (2011) Mirror neuron forum. Perspectives on Psychological Science 6: 369–407. 25520744 10.1177/1745691611413392 PMC4266473

[26] Rizzolatti G, Arbib MA (1998) Language within our grasp. Trends Neurosci 21: 188–194. 9610880 10.1016/s0166-2236(98)01260-0

[27] Golumbic EM, Poeppel D, Schroeder CE (2012) Temporal context in speech processing and attentional stream selection: A behavioral and neural perspective. Brain & Language, 122, 151–161. 10.1016/j.bandl.2011.12.010 PMC3340429 22285024

[28] Arbib MA (2006) Action to language via the mirror neuron system. Cambridge University Press. 567 p.

[29] Callan DE, Jones JA, Munhall K, Callan AM, Kroos C, et al (2003) Neural processes underlying perceptual enhancement by visual speech gestures. Neuroreport 14: 2213. 14625450 10.1097/00001756-200312020-00016

[30] Rauschecker JP, Scott SK (2009) Maps and streams in the auditory cortex: nonhuman primates illuminate human speech processing. Nature neuroscience 12: 718–724. 19471271 10.1038/nn.2331 PMC2846110

[31] Hickok G, Poeppel D (2000) Towards a functional neuroanatomy of speech perception. Trends in Cognitive Sciences 4: 131–138. 10740277 10.1016/s1364-6613(00)01463-7

[32] Arbib MA (2010) Mirror system activity for action and language is embedded in the integration of dorsal and ventral pathways. Brain and Language 112: 12–24. 19942271 10.1016/j.bandl.2009.10.001

[33] Guenther FH, Perkell JS (2004) A neural model of speech production and its application to studies of the role of auditory feedback in speech. Speech motor control in normal and disordered speech: 29–49.

[34] Callan DE, Kent RD, Guenther FH, Vorperian HK (2000) An auditory-feedback-based neural network model of speech production that is robust to developmental changes in the size and shape of the articulatory system. J Speech Lang Hear Res 43: 721–736. 10877441 10.1044/jslhr.4303.721

[35] Hasson U, Skipper JI, Nusbaum HC, Small SL (2007) Abstract coding of audiovisual speech: Beyond sensory representation. Neuron 56: 1116–1126. 18093531 10.1016/j.neuron.2007.09.037 PMC2175551

[36] Poeppel D, Idsardi WJ, Van Wassenhove V (2008) Speech perception at the interface of neurobiology and linguistics. Philosophical Transactions of the Royal Society B: Biological Sciences 363: 1071–1086. 10.1098/rstb.2007.2160 PMC2606797 17890189

[37] Van Wassenhove V, Grant KW, Poeppel D (2005) Visual speech speeds up the neural processing of auditory speech. Proceedings of the National Academy of Sciences of the United States of America 102: 1181–1186. 15647358 10.1073/pnas.0408949102 PMC545853

[38] Zatorre RJ, Evans AC, Meyer E, Gjedde A (1992) Lateralization of phonetic and pitch discrimination in speech processing. Science 256: 846–849. 1589767 10.1126/science.1589767

[39] Burton MW, Noll DC, Small SL (2001) The anatomy of auditory word processing: individual variability. Brain Lang 77: 119–131. 11247659 10.1006/brln.2000.2444

[40] Binder JR, Liebenthal E, Possing ET, Medler DA, Ward BD (2004) Neural correlates of sensory and decision processes in auditory object identification. Nature Neuroscience 7: 295–301. 14966525 10.1038/nn1198

[41] Callan DE, Jones JA, Callan AM, Akahane-Yamada R (2004) Phonetic perceptual identification by native- and second-language speakers differentially activates brain regions involved with acoustic phonetic processing and those involved with articulatory-auditory/orosensory internal models. Neuroimage 22: 1182–1194. 15219590 10.1016/j.neuroimage.2004.03.006

[42] Meister IG, Wilson SM, Deblieck C, Wu AD, Iacoboni M (2007) The essential role of premotor cortex in speech perception. Curr Biol 17: 1692–1696. 17900904 10.1016/j.cub.2007.08.064 PMC5536895

[43] D’Ausilio A, Pulvermüller F, Salmas P, Bufalari I, Begliomini C, et al (2009) The motor somatotopy of speech perception. Curr Biol 19: 381–385. 19217297 10.1016/j.cub.2009.01.017

[44] Sato M, Tremblay P, Gracco VL (2009) A mediating role of the premotor cortex in phoneme segmentation. Brain Lang 111: 1–7. 19362734 10.1016/j.bandl.2009.03.002

[45] LoCasto PC, Krebs-Noble D, Gullapalli RP, Burton MW (2004) An fMRI investigation of speech and tone segmentation. J Cogn Neurosci 16: 1612–1624. 15601523 10.1162/0898929042568433

[46] Joanisse MF, Gati JS (2003) Overlapping neural regions for processing rapid temporal cues in speech and nonspeech signals. Neuroimage 19: 64–79. 12781727 10.1016/s1053-8119(03)00046-6

[47] Burton M (2009) Understanding the role of the prefrontal cortex in phonological processing. Clinical Linguistics & Phonetics 23: 180–195. 19283576 10.1080/02699200802394963

[48] Le Bel RM, Pineda JA, Sharma A (2009) Motor-auditory-visual integration: The role of the human mirror neuron system in communication and communication disorders. Journal of communication disorders 42: 299–304. 19419735 10.1016/j.jcomdis.2009.03.011 PMC2770869

[49] Max L, Guenther FH, Gracco VL, Ghosh SS, Wallace ME (2004) Unstable or insufficiently activated internal models and feedback-biased motor control as sources of dysfluency: A theoretical model of stuttering. http://citeseerx.ist.psu.edu/viewdoc/summary doi = 10.1.1.4.3841.

[50] Vanni S, Portin K, Virsu V, Hari R (1999) Mu rhythm modulation during changes of visual percepts. Neuroscience 91: 21–31. 10336056 10.1016/s0306-4522(98)00521-1

[51] Nagamine T, Kajola M, Salmelin R, Shibasaki H, Hari R (1996) Movement-related slow cortical magnetic fields and changes of spontaneous MEG- and EEG-brain rhythms. Electroencephalogr Clin Neurophysiol 99: 274–286. 8862117 10.1016/0013-4694(96)95154-8

[52] Nishitani N, Hari R (2000) Temporal dynamics of cortical representation for action. Proceedings of the National Academy of Sciences of the United States of America 97: 913–918. 10639179 10.1073/pnas.97.2.913 PMC15430

[53] Nishitani N, Hari R (2002) Viewing lip forms: cortical dynamics. Neuron 36: 1211–1220. 12495633 10.1016/s0896-6273(02)01089-9

[54] Möttönen R, Järveläinen J, Sams M, Hari R (2005) Viewing speech modulates activity in the left SI mouth cortex. Neuroimage 24: 731–737. 15652308 10.1016/j.neuroimage.2004.10.011

[55] Oberman LM, Hubbard EM, McCleery JP, Altschuler EL, Ramachandran VS, et al (2005) EEG evidence for mirror neuron dysfunction in autism spectrum disorders. Brain Res Cogn Brain Res 24: 190–198. 15993757 10.1016/j.cogbrainres.2005.01.014

[56] Oberman LM, Ramachandran VS, Pineda JA (2008) Modulation of mu suppression in children with autism spectrum disorders in response to familiar or unfamiliar stimuli: the mirror neuron hypothesis. Neuropsychologia 46: 1558–1565. 18304590 10.1016/j.neuropsychologia.2008.01.010

[57] Ulloa ER, Pineda JA (2007) Recognition of point-light biological motion: mu rhythms and mirror neuron activity. Behav Brain Res 183: 188–194. 17658625 10.1016/j.bbr.2007.06.007

[58] Pineda JA (2008) Sensorimotor cortex as a critical component of an “extended” mirror neuron system: Does it solve the development, correspondence, and control problems in mirroring? Behav Brain Funct 4: 47. 18928566 10.1186/1744-9081-4-47 PMC2577683

[59] Pineda JA, Allison BZ, Vankov A (2000) The effects of self-movement, observation, and imagination on mu rhythms and readiness potentials (RP’s): toward a brain-computer interface (BCI). IEEE Trans Rehabil Eng 8: 219–222. 10896193 10.1109/86.847822

[60] Muthukumaraswamy SD, Johnson BW, McNair NA (2004) Mu rhythm modulation during observation of an object-directed grasp. Brain Res Cogn Brain Res 19: 195–201. 15019715 10.1016/j.cogbrainres.2003.12.001

[61] Crawcour S, Bowers A, Harkrider A, Saltuklaroglu T (2009) Mu wave suppression during the perception of meaningless syllables: EEG evidence of motor recruitment. Neuropsychologia 47: 2558–2563. 19442676 10.1016/j.neuropsychologia.2009.05.001

[62] Muthukumaraswamy SD, Johnson BW (2004) Primary motor cortex activation during action observation revealed by wavelet analysis of the EEG. Clin Neurophysiol 115: 1760–1766. 15261854 10.1016/j.clinph.2004.03.004

[63] Hari R (2006) Action–perception connection and the cortical mu rhythm. Progress in Brain Research 159: 253–260. 17071236 10.1016/S0079-6123(06)59017-X

[64] Jensen O, Goel P, Kopell N, Pohja M, Hari R, et al (2005) On the human sensorimotor-cortex beta rhythm: sources and modeling. Neuroimage 26: 347–355. 15907295 10.1016/j.neuroimage.2005.02.008

[65] Pineda JA (2005) The functional significance of mu rhythms: translating “seeing” and “hearing” into “doing”. Brain Res Brain Res Rev 50: 57–68. 15925412 10.1016/j.brainresrev.2005.04.005

[66] Cuellar M, Bowers A, Harkrider AW, Wilson M, Saltuklaroglu T (2012) Mu suppression as an index of sensorimotor contributions to speech processing: Evidence from continuous EEG signals. International Journal of Psychophysiology 85: 242–248. 22522370 10.1016/j.ijpsycho.2012.04.003

[67] Onton J, Makeig S (2006) Information-based modeling of event-related brain dynamics. Event-Related Dynamics of Brain Oscillations. Elsevier, Vol. Volume 159: 99–120. 10.1016/S0079-6123(06)59007-7 17071226

[68] Moore A, Gorodnitsky I, Pineda J (2012) EEG mu component responses to viewing emotional faces. Behavioural brain research 226: 309–316. 21835208 10.1016/j.bbr.2011.07.048

[69] Oldfield RC (1971) The assessment and analysis of handedness: the Edinburgh inventory. Neuropsychologia 9: 97–113. 5146491 10.1016/0028-3932(71)90067-4

[70] Ernst SMA, Verhey JL, Uppenkamp S (2008) Spatial dissociation of changes of level and signal-to-noise ratio in auditory cortex for tones in noise. NeuroImage 43: 321–328. 18722535 10.1016/j.neuroimage.2008.07.046

[71] Graimann B, Pfurtscheller G (2006) Quantification and visualization of event-related changes in oscillatory brain activity in the time-frequency domain. Prog Brain Res 159: 79–97. 17071225 10.1016/S0079-6123(06)59006-5

[72] Makeig S, Delorme A, Westerfield M, Jung TP, Townsend J, et al (2004) Electroencephalographic brain dynamics following manually responded visual targets. PLoS biology 2: e176. 15208723 10.1371/journal.pbio.0020176 PMC423146

[73] Jasper,H.A (1958) The ten–twenty system of the International Federation. Electroencepholography and Clinical Neurophysiology 10: 371–375.

[74] Congedo M, John R, De Ridder D, Prichep L, Isenhart R (2009) On the dependence of independent group EEG sources; an EEG study on two large databases. 10.1007/s10548-009-0113-6 19802727

[75] White D, Congedo M, Ciociari J, Silberstein R (2012) Brain oscillatory activity during spatial navigation : Theta and gamma activity link medial temporal and parietal regions. Journal of Cognitive Neuroscience 3: 686–697. 10.1162/jocn_a_00098 21812639

[76] Lee TW, Girolami M, Sejnowski TJ (1999) Independent component analysis using an extended infomax algorithm for mixed subgaussian and supergaussian sources. Neural computation 11: 417–441. 9950738 10.1162/089976699300016719

[77] Mognon A, Jovicich J, Bruzzone L, Buiatti M (2010) ADJUST: An automatic EEG artifact detector based on the joint use of spatial and temporal features. Psychophysiology. 10.1111/j.1469-8986.2010.01061.x 20636297

[78] Oostenveld R, Oostendorp TF (2002) Validating the boundary element method for forward and inverse EEG computations in the presence of a hole in the skull. Human brain mapping 17: 179–192. 12391571 10.1002/hbm.10061 PMC6872070

[79] Pascual-Marqui RD (2002) Standardized low-resolution brain electromagnetic tomography (sLORETA): technical details. Methods Find Exp Clin Pharmacol 24 Suppl D: 5–12. 12575463

[80] Towle VL, Bolaños J, Suarez D, Tan K, Grzeszczuk R, et al (1993) The spatial location of EEG electrodes: locating the best-fitting sphere relative to cortical anatomy. Electroencephalography and Clinical Neurophysiology 86: 1–6. 7678386 10.1016/0013-4694(93)90061-y

[81] Delorme A (2004) EEGLAB: an open source toolbox for analysis of single-trial EEG dynamics including independent component analysis. Journal of neuroscience methods 134: 9–21. 15102499 10.1016/j.jneumeth.2003.10.009

[82] Benjamini Y, Hochberg Y (2001) On the adaptive control of the false discovery rate in multiple testing with independent statistics. Journal of Educational and Behavioral Statistics 25: 60–83.

[83] Grin-Yatsenko VA, Baas I, Ponomarev VA, Kropotov JD (2010) Independent component approach to the analysis of EEG recordings at early stages of depressive disorders. Clinical Neurophysiology 121: 281–289. 20006545 10.1016/j.clinph.2009.11.015

[84] Vanni S, Rockstroh B, Hari R (1996) Cortical sources of human short-latency somatosensory evoked fields to median and ulnar nerve stimuli. Brain Res 737: 25–33. 8930346 10.1016/0006-8993(96)00646-4

[85] Salenius S, Hari R (2003) Synchronous cortical oscillatory activity during motor action. Current opinion in neurobiology 13: 678–684. 14662368 10.1016/j.conb.2003.10.008

[86] Jasper H, Penfield W (1949) Electrocorticograms in man: effect of voluntary movement upon the electrical activity of the precentral gyrus. European Archives of Psychiatry and Clinical Neuroscience 183: 163–174.

[87] Conway BA, Halliday DM, Farmer SF, Shahani U, Maas P, et al (1995) Synchronization between motor cortex and spinal motoneuronal pool during the performance of a maintained motor task in man. The Journal of physiology 489: 917–924. 8788955 10.1113/jphysiol.1995.sp021104 PMC1156860

[88] Press C, Cook J, Blakemore SJ, Kilner J (2012) Dynamic modulation of human motor activity when observing actions. J Neuroci 8: 2792–2800. 10.1523/JNEUROSCI.1595-10.2011 PMC3398132 21414901

[89] Avanzini P, Maddalena FD, Riccardo DV, Daprati E, Rizzolatti G, Gaetano C (2012) PloS One 5.

[90] Jensen O, Goel P, Kopell N, Pohja M, Hari R, et al (2005) On the human sensorimotor-cortex beta rhythm: sources and modeling. Neuroimage 26: 347–355. 15907295 10.1016/j.neuroimage.2005.02.008

[91] Michels L, Bucher K, Lüchinger R, Klaver P, Martin E, et al (2010) Simultaneous EEG-fMRI during a working memory task: modulations in low and high frequency bands. PLoS One 5: e10298. 20421978 10.1371/journal.pone.0010298 PMC2858659

[92] Perry A, Bentin S (2009) Mirror activity in the human brain while observing hand movements: a comparison between EEG desynchronization in the mu-range and previous fMRI results. Brain Res 1282: 126–132. 19500557 10.1016/j.brainres.2009.05.059

[93] Ritter P, Moosmann M, Villringer A (2009) Rolandic alpha and beta EEG rhythms’ strengths are inversely related to fMRI-BOLD signal in primary somatosensory and motor cortex. Human brain mapping 30: 1168–1187. 18465747 10.1002/hbm.20585 PMC6870597

[94] Yuan H, Liu T, Szarkowski R, Rios C, Ashe J, et al (2010) Negative covariation between task-related responses in alpha/beta-band activity and BOLD in human sensorimotor cortex: An EEG and fMRI study of motor imagery and movements. Neuroimage 49: 2596–2606. 19850134 10.1016/j.neuroimage.2009.10.028 PMC2818527

[95] Meister IG, Wilson SM, Deblieck C, Wu AD, Iacoboni M (2007) The essential role of premotor cortex in speech perception. Curr Biol 17: 1692–1696. 17900904 10.1016/j.cub.2007.08.064 PMC5536895

[96] Callan DE, Tajima K, Callan AM, Kubo R, Masaki S, et al (2003) Learning-induced neural plasticity associated with improved identification performance after training of a difficult second-language phonetic contrast. Neuroimage 19: 113–124. 12781731 10.1016/s1053-8119(03)00020-x

[97] Wilson SM, Saygin AP, Sereno MI, Iacoboni M (2004) Listening to speech activates motor areas involved in speech production. Nat Neurosci 7: 701–702. 15184903 10.1038/nn1263

[98] Alho J, Sato M, Sams M, Schwartz JL, Tiitinen H, et al (2012) Enhanced early-latency electromagnetic activity in the left premotor cortex is associated with successful phonetic categorization. NeuroImage. 60: 1937–1946. 10.1016/j.neuroimage.2012.02.011 22361165

[99] Callan DE, Tsytsarev V, Hanakawa T, Callan AM, Katsuhara M, et al (2006) Song and speech: brain regions involved with perception and covert production. Neuroimage 31: 1327–1342. 16546406 10.1016/j.neuroimage.2006.01.036

[100] Zatorre RJ, Belin P, Penhune VB (2002) Structure and function of auditory cortex: music and speech. Trends in Cognitive Sciences 6: 37–46. 11849614 10.1016/s1364-6613(00)01816-7

[101] Hickok G, Buchsbaum B, Humphries C, Muftuler T (2003) Auditory-motor interaction revealed by fMRI: speech, music, and working memory in area Spt. Journal of Cognitive Neuroscience 15: 673–682. 12965041 10.1162/089892903322307393

[102] Dick F, Lee HL, Nusbaum H, Price CJ (2011) Auditory-motor expertise alters “speech selectivity” in professional musicians and actors. Cerebral Cortex 21: 938–948. 20829245 10.1093/cercor/bhq166 PMC3059891

[103] Basar E, Schurmann M, Basar-Eroglu C, Karakas S (1997) Alpha oscillations in brain functioning: an integrative theory. International Journal of Psychophysiology 26: 5–29. 9202992 10.1016/s0167-8760(97)00753-8

[104] Fellinger R, Klimesch W, Gruber W, Freunberger R, Doppelmayr M (2011) Pre-stimulus alpha phase-alignment predicts P1-amplitude. Brain Res Bull 85: 417–423. 21473900 10.1016/j.brainresbull.2011.03.025 PMC3144391

[105] Fellinger R, Klimesch W, Schnakers C, Perrin F, Freunberger R, et al (2011) Cognitive processes in disorders of consciousness as revealed by EEG time-frequency analyses. Clin Neurophysiol 122: 2177–2184. 21511524 10.1016/j.clinph.2011.03.004

[106] Fellinger R, Gruber W, Zauner A, Freunberger R, Klimesch W (2012) Evoked traveling alpha waves predict visual-semantic categorization-speed. Neuroimage 59: 3379–3388. 22100769 10.1016/j.neuroimage.2011.11.010 PMC3314919

[107] Freunberger R, Fellinger R, Sauseng P, Gruber W, Klimesch W (2009) Dissociation between phase-locked and nonphase-locked alpha oscillations in a working memory task. Hum Brain Mapp 30: 3417–3425. 19384888 10.1002/hbm.20766 PMC6870638

[108] Hoedlmoser K, Griessenberger H, Fellinger R, Freunberger R, Klimesch W, et al (2011) Event-related activity and phase locking during a psychomotor vigilance task over the course of sleep deprivation. J Sleep Res 20: 377–385. 20977513 10.1111/j.1365-2869.2010.00892.x PMC3439125

[109] Klimesch W, Fellinger R, Freunberger R (2011) Alpha oscillations and early stages of visual encoding. Front Psychol 2: 118. 21687470 10.3389/fpsyg.2011.00118 PMC3108577

[110] Marco-Pallarés J, Grau C, Ruffini G (2005) Combined ICA-LORETA analysis of mismatch negativity. Neuroimage 25: 471–477. 15784426 10.1016/j.neuroimage.2004.11.028

[111] Kohler E, Keysers C, Umiltà MA, Fogassi L, Gallese V, et al (2002) Hearing sounds, understanding actions: action representation in mirror neurons. Science 297: 846–848. 12161656 10.1126/science.1070311

[112] Arnal L, Giraud AL (2012) Cortical Oscillations and Sensory Predictions. Cell Press: 1–9. 10.1016/j.tics.2012.05.003 22682813

[113] Galantucci B, Fowler CA, Turvey MT (2006) The motor theory of speech perception reviewed. Psychon Bull Rev 13: 361–377. 17048719 10.3758/bf03193857 PMC2746041

[114] Szenkovits G, Peelle JE, Norris D, Davis MH (2012) Individual differences in premotor and motor recruitment during speech perception. Neuropsychologia 50: 1380–1392. 22521874 10.1016/j.neuropsychologia.2012.02.023

[115] Wong P, Jin JX, Gunasekera GM, Abel R, Lee ER, et al (2009) Aging and cortical mechanisms of speech perception in noise. Neuropsychologia 47: 693–703. 19124032 10.1016/j.neuropsychologia.2008.11.032 PMC2649004

[116] Borrie SA, McAuliffe MJ, Liss JM (2012) Perceptual learning of dysarthric speech: A review of experimental studies. Journal of Speech, Language and Hearing Research 55: 290. 10.1044/1092-4388(2011/10-0349) PMC3738172 22199185

[117] Mottonen R, Watkins KE (2012) Using TMS to study the role of the articulatory motor system in speech perception,” *Aphasiology*, 26, 1103–1118. 10.1080/02687038.2011.619515 PMC3431548 22942513

[118] Debener S, Ullsperger M, Siegel M, Engel AK (2006) Single-trial EEG-fMRI reveals the dynamics of cognitive function. Trends in cognitive sciences 10: 558–563. 17074530 10.1016/j.tics.2006.09.010

